# Histone-like nucleoid-structuring protein (H-NS) regulatory role in antibiotic resistance in *Acinetobacter baumannii*

**DOI:** 10.1038/s41598-021-98101-w

**Published:** 2021-09-16

**Authors:** Deja Rodgers, Casin Le, Camila Pimentel, Marisel R. Tuttobene, Tomás Subils, Jenny Escalante, Brent Nishimura, Eleonora García Vescovi, Rodrigo Sieira, Robert A. Bonomo, Marcelo E. Tolmasky, Maria Soledad Ramirez

**Affiliations:** 1 Center for Applied Biotechnology Studies, Department of Biological Science, College of Natural Sciences and Mathematics, California State University Fullerton, 800 N State College Blvd, Fullerton, CA 92831 USA; 2 Área Biología Molecular, Facultad de Ciencias Bioquímicas y Farmacéuticas, Universidad Nacional de Rosario, Rosario, Argentina; 3 Instituto de Biología Molecular y Celular de Rosario (IBR, CONICET-UNR), Rosario, Argentina; 4 Instituto de Procesos Biotecnológicos y Químicos de Rosario (IPROBYQ, CONICET-UNR), Rosario, Argentina; 5 Fundación Instituto Leloir – IIBBA CONICET, Buenos Aires, Argentina; 6 Research Service and GRECC, Department of Veterans Affairs Medical Center, Louis Stokes Cleveland, Cleveland, OH USA; 7 Departments of Medicine, Pharmacology, Molecular Biology and Microbiology, Biochemistry, Proteomics and Bioinformatics, Case Western Reserve University School of Medicine, Cleveland, OH USA; 8 CWRU-Cleveland VAMC Center for Antimicrobial Resistance and Epidemiology (Case VA CARES), Cleveland, OH USA

**Keywords:** Microbiology, Antimicrobials, Biofilms, Pathogens

## Abstract

In the multidrug resistant (MDR) pathogen *Acinetobacter baumannii* the global repressor H-NS was shown to modulate the expression of genes involved in pathogenesis and stress response. In addition, H-NS inactivation results in an increased resistance to colistin, and in a hypermotile phenotype an altered stress response. To further contribute to the knowledge of this key transcriptional regulator in *A. baumannii* behavior, we studied the role of H-NS in antimicrobial resistance. Using two well characterized *A. baumannii* model strains with distinctive resistance profile and pathogenicity traits (AB5075 and A118), complementary transcriptomic and phenotypic approaches were used to study the role of H-NS in antimicrobial resistance, biofilm and quorum sensing gene expression. An increased expression of genes associated with β-lactam resistance, aminoglycosides, quinolones, chloramphenicol, trimethoprim and sulfonamides resistance in the *Δhns* mutant background was observed. Genes codifying for efflux pumps were also up-regulated, with the exception of *adeFGH*. The wild-type transcriptional level was restored in the complemented strain. In addition, the expression of biofilm related genes and biofilm production was lowered when the transcriptional repressor was absent. The quorum network genes *aidA, abaI, kar* and *fadD* were up-regulated in *Δhns* mutant strains. Overall, our results showed the complexity and scope of the regulatory network control by H-NS (genes involved in antibiotic resistance and persistence). These observations brings us one step closer to understanding the regulatory role of *hns* to combat *A. baumannii* infections.

## Introduction

Transcription, as a complex obligated process for gene expression, provides multiple targets for antimicrobial discovery. Among them, obvious candidates are transcriptional factors and regulators. While RNA polymerase and transcriptional factors are highly conserved across the bacterial kingdom, regulators can be more specific to each bacterium that must adapt to unique environments. *Acinetobacter baumannii* readily adjusts to the hostile environments it finds when it colonizes and infects different human body niches. A critical aspect of the adaptation strategies to survive and thrive in harsh conditions is the fine tuning of gene expression through the timely action of transcriptional regulators. Researchers have explored the regulation of antibiotic resistance and identified several two-component systems (TCSs) responsible for different mechanisms such as regulation of RND-type efflux pumps^1–4^.

A transcription factor of interest is H-NS, since it controls expression of many functions in bacteria, including adherence to biotic surfaces, quorum sensing, biofilm formation, and metabolism pathways^5–9^. In *A. baumannii*, H-NS regulates the expression of genes involved in a variety of biological processes like transport (e.g., autotransporters, type-VI secretion systems) and metabolism (e.g., phenylacetic acid degradation pathway)^7^. Interestingly, H-NS inactivation, reduces levels of resistance to colistin^8^, an antibiotic currently used as a last resource to treat multidrug resistant (MDR) *A. baumannii* infections. H-NS is also responsible for protecting against stress; it alleviates the deleterious effects produced by metallo-β-lactamase toxic precursors and protects against DNA-damaging agents such as mitomycin C and levofloxacin^10^. A recently described function of H-NS is the repression of the exopolysaccharide poly-N-acetylglucosamine synthesis and the expression of a protein belonging to the CsgG/HfaB family^11^. The expression of the *A. baumannii* H-NS is down regulated in environments containing human serum albumin (HSA) or HSA-containing fluids^12,13^. In sum, the regulatable nature of H-NS strongly suggests it plays a fundamental role in *A. baumannii’*s responses and adaptation to the conditions within the human body.

In this study, we demonstrate the role of H-NS in regulating expression of genes related to antibiotic resistance, biofilm formation, and quorum sensing in the multidrug-resistant, hypervirulent *A. baumannii* AB5075 and the susceptible, moderately virulent *A. baumannii* A118 strains.

## Results and discussion

Transcriptome analysis of *A. baumannii* strain AB5075 and AB5075 *Δhns* revealed a differential expression profile of 183 genes with an FDR-adjusted *P-value* < 0.05 and log2-fold change > 1. Among the differentially expressed-genes (DEGs), we identified genes associated with motility, biofilm formation, quorum sensing, antibiotic resistance, metabolism, and stress response (Supplementary Table S1). As the aim of the present work is to identify the possible role of H-NS in antibiotic resistance only those genes, directly or indirectly associated with antibiotic resistance were examined (Supplementary Table S2).

### H-NS plays a role in modulating the expression of antibiotic resistance associated genes

**Figure 1:**
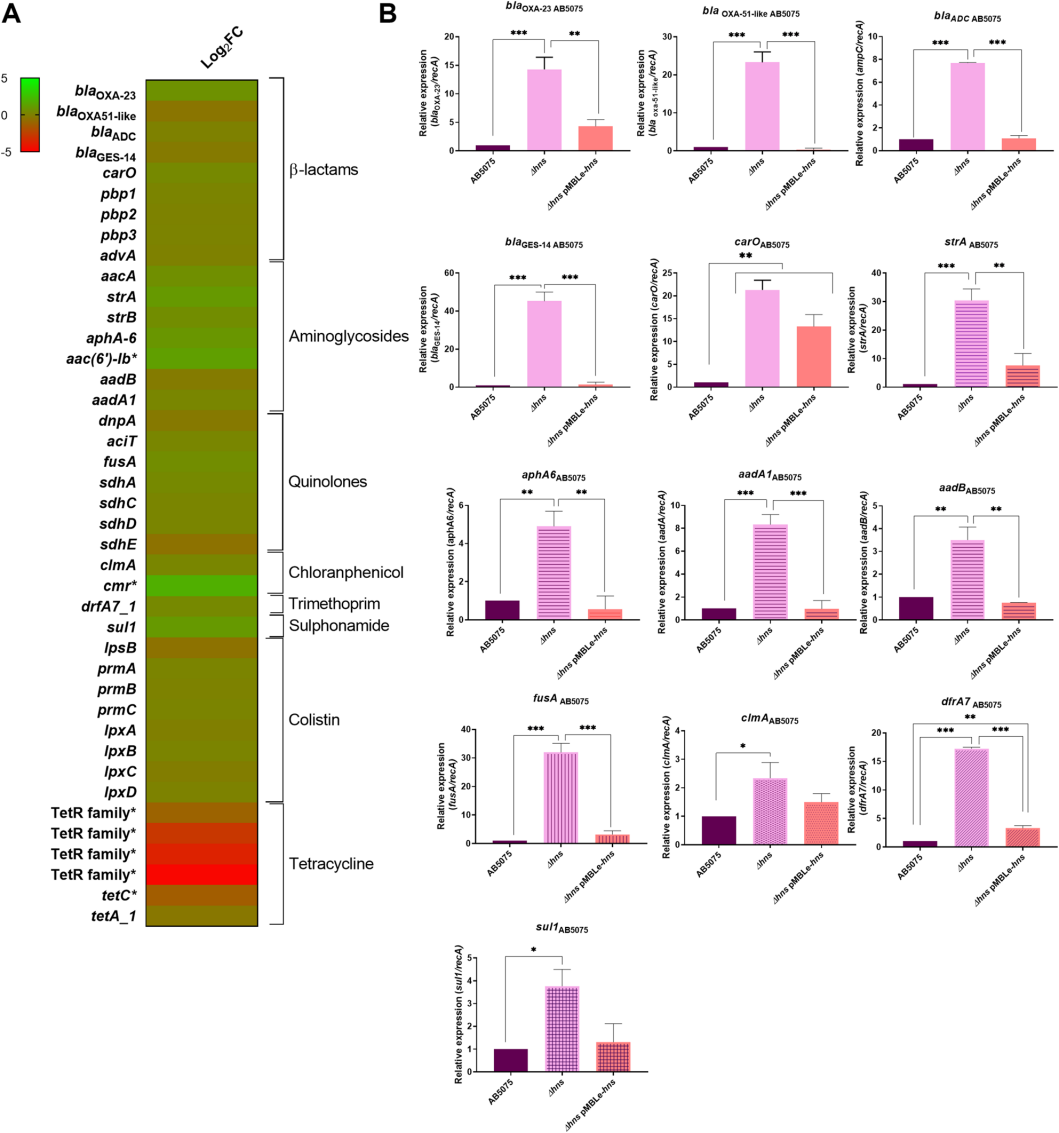
(**A**) Heatmap outlining the differential expression of genes associated with antibiotic resistance in *A. baumannii* AB5075 *Δhns* vs. parental strain. The asterisks represent the DEGs (adjusted *P*-value < 0.05 with log2fold change > 1). Heat map was performed using GraphPad Prism version number 9 (GraphPad software, San Diego, CA, USA, https://www.graphpad.com/). (**B**) qRT-PCR of AB5075, AB5075 *Δhns* and AB5075 *Δhns* pMBLe-*hns* strains genes associated with antibiotic resistance, the expression levels and the fold changes were determinate using the double ΔCt analysis. At least three independent samples were used, and three technical replicates were performed from each sample. Statistical significance (*P*-value < 0.05) was determined by ANOVA followed by Tukey’s multiple comparison test; one asterisks: *P*-value < 0.05; two asterisks: *P*-value < 0.01 and three asterisks: *P*-value < 0.001. Different patterns in the graphs indicate different groups of genes associated with antibiotic resistance. This figure was performed using GraphPad Prism version number 9 (GraphPad software, San Diego, CA, USA, https://www.graphpad.com/).

Analyzed RNA-seq data showed that the expression of genes related to resistance to β-lactams, aminoglycosides, quinolones, chloramphenicol, trimethoprim, sulfonamides, and colistin were up-regulated in AB5075 *Δhns* strain (Fig. 1A). On the other hand, genes related to tetracycline resistance were down-regulated in the *Δhns* strain compared to the parental strain. To confirm the RNA-seq results, transcript analysis of *A. baumannii* AB5075, AB5075 *Δhns* and AB5075 *Δhns* pMBLe-*hns* cells was performed, showing an enhanced expression on seven out of eight genes (*bla*_OXA-23_, *bla*_OXA-51-like_, *bla*_ADC_, *bla*_GES-14_, *carO, pbp1*, and *advA*) associated with β-lactam resistance in AB5075 *Δhns* mutant strain (Fig. 1B and Supplementary Fig. S1A). In addition, the increased expression of all the selected genes associated with aminoglycoside, quinolone, chloramphenicol, trimethoprim and sulfonamide resistance in AB5075 *Δhns* strain was confirmed by quantitative RT-PCR (qRT-PCR) assays (Fig. 1B and Supplementary Fig. S1A). Consistently, the mutant strain containing the plasmid pMBLe-*hns*, which expresses a wild-type copy of *hns* under the control of its own promoter, rescued the expression for all resistance-related genes tested to wild type levels (Fig. 1B). These results provide evidence indicating that H-NS is involved in modulating the expression of resistance-related genes.

In order to further expand our knowledge about antibiotic resistance regulation, qRT-PCR experiments were carried out using the susceptible and less virulent *A. baumannii* A118 strain. This isolated was selected since it is an *A. baumannii* representative strains that we and other authors have been using in previous works as a model strain^11–24^. This strain belongs to a different lineages respect to AB5075 strain, A118 is a sporadic clone while AB5075 is a representative strain of one of the most prevalent clones (GC1). We observed that, four out of five (*bla*_OXA-51-like_*, pbp1, pbp3, and carO*) genes associated with β-lactam resistance as well as the fluoroquinolone resistance gene *fusA* were up-regulated (Supplementary Fig. S1B).

To test the effect of H-NS on the antibiotic susceptibility profile of *A. baumannii* strains, disk diffusion and minimal inhibitory concentration (MIC) measurements were performed (Supplementary Tables S3 and S4 and Supplementary Fig. S2). Observing disk diffusion, an increase in the halo of 4 mm or more was observed for meropenem, imipenem, amikacin and gentamicin for both strains, considering significantly according to CLSI breakpoints (Supplementary Table S3). MICs revealed twofold, threefold, threefold and fivefold decreases in AB5075 Δ*hns* for meropenem, imipenem, amikacin and gentamicin, respectively (Supplementary Table S4 and Supplementary Fig. S2), changing from resistant to susceptible for gentamicin and amikacin, and resistant to intermediate for imipenem. In the A118 strain, a highly susceptible strain, slight changes in MICs values were observed in the Δ*hns* strain for the carbapenems antibiotics (Supplementary Table S4). However, significant fold-decreases were observed for amikacin and gentamicin MICs (Supplementary Table S4). Changes in MIC were not observed for ceftazidime, ciprofloxacin, norfloxacin, and colistin for both strains (Table S4).

Overall, an increase in expression of the analyzed antibiotic resistance genes is observed in *Δhns* mutant strains suggesting a pleiotropic modulation of gene expression. The increased expression of some genes cannot be correlated with the respective phenotype, such as the increase expression of *aphA6* and *aadB* and the increase in susceptibility for amikacin and gentamicin, respectively. However, because antibiotic resistance mechanisms are multifactorial and other uncharacterized or unknown genes might be involved, H-NS could be partially responsible for the observed phenotypes.

A previous analysis using metallo-β-lactamases (MBLs) and DNA-damaging agents as stressors^9^, showed that the AB5075 *Δhns* strain showed diminished ability to overcome stress^9^. Moreover, imipenem MICs of strains expressing the different MBLs, showed a detrimental impact on the antibiotic resistance phenotype in the Δ*hns* background^9^. In addition, the increased susceptibility to imipenem observed in both mutant strains can be explained in part by the significant increase in expression of *carO* (Supplementary Table S4, and Fig. 1 and Supplementary Fig. S1), a correlation between the expression of CarO and the outer membrane permeation to imipenem in *A. baumannii* has been reported^25,26^.

The reduced MICs observed in the present work for carbapenems and aminoglycosides in the mutant background, could be related with the role of H-NS in stress response. In accordance with this result, RNA-seq analysis of the AB5075 mutant strain showed the regulation of genes that encode for proteins involved in counteracting stressful conditions, such as *cspE, cspG, ywrO, hsp40, slt, mltB, katE, soxR* and *soxS* genes (Supplementary Table S1). Diverse stress conditions, such as oxidative stress, mediated by global regulatory genes like SoxR, which is a global repressor protein involved in multidrug-resistance in Gram negative bacteria, cause the overexpression of efflux pumps contributing to multidrug-resistance phenotype ^27^. A recent in vitro study showed that under consecutive imipenem stress, *A. baumannii* evolved the ability to reduce susceptibility to various antimicrobials, including carbapenem, through overproduction of the RND super-family efflux pump ^28^. In other Gram-negative bacteria, as in *Vibrio cholerae*, it was shown that the loss of H-NS is involved in inducing an endogenous envelope/oxidative stress ^29^.

**Figure 2:**
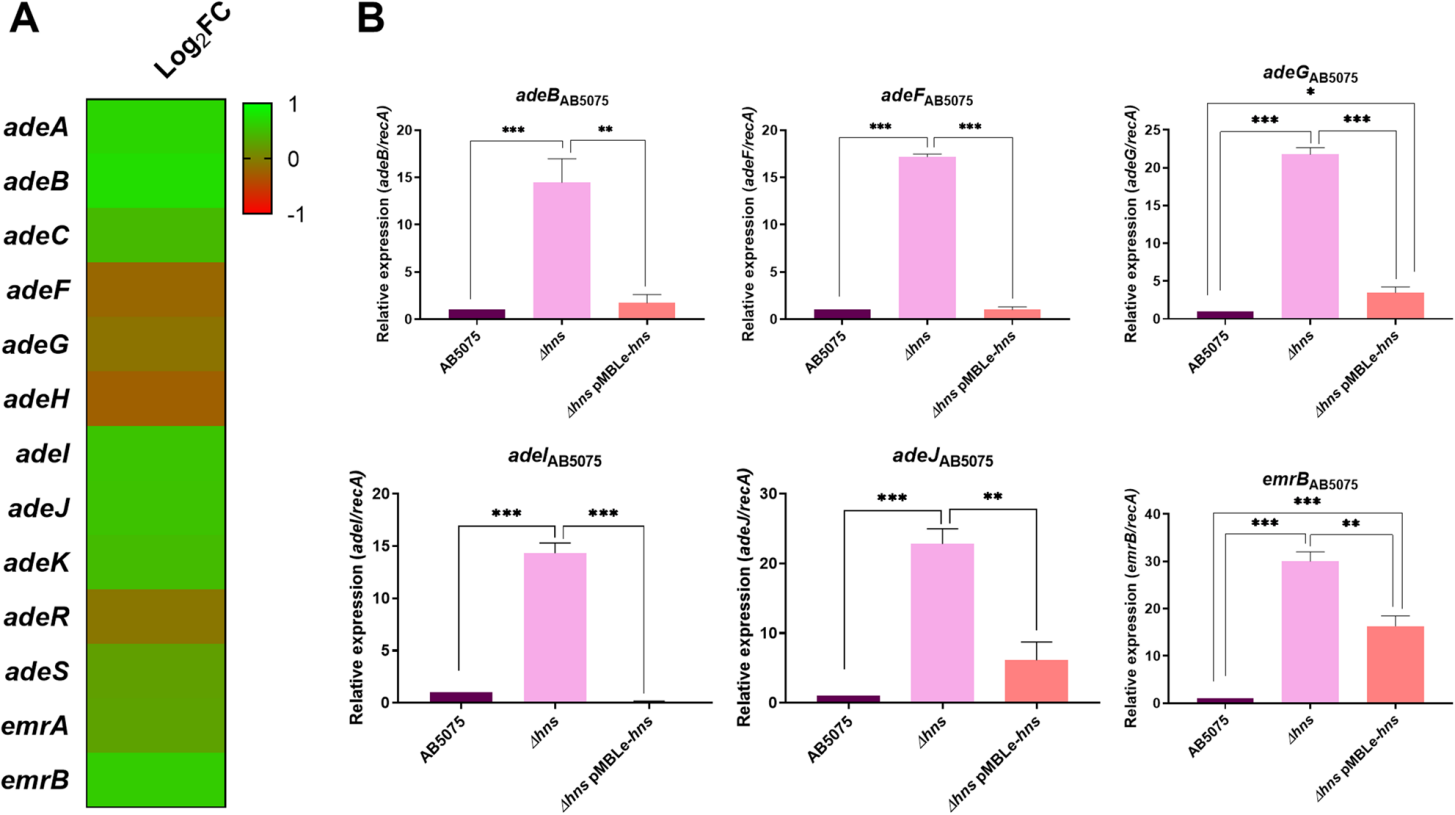
Genetic analysis of efflux pumps coding genes. (**A**) Heatmap outlining the differential expression of genes associated with efflux pumps in *A. baumannii* AB5075 *Δhns* vs. parental strain. The asterisks represent the DEGs (adjusted *P*-value < 0.05 with log2fold change > 1). Heat map was performed using GraphPad Prism version number 9 (GraphPad software, San Diego, CA, USA, https://www.graphpad.com/). (**B**) qRT-PCR of AB5075, AB5075 *Δhns* and AB5075 *Δhns* pMBLe-*hns* strains genes associated with efflux pumps, the expression levels and the fold changes were determinate using the double ΔCt analysis. At least three independent samples were used, and three technical replicates were performed from each sample. Statistical significance (*P*-value < 0.05) was determined by ANOVA followed by Tukey’s multiple comparison test; one asterisks: *P*-value < 0.05; two asterisks: *P*-value < 0.01 and three asterisks: *P*-value < 0.001. This figure was performed using GraphPad Prism version number 9 (GraphPad software, San Diego, CA, USA, https://www.graphpad.com/).

To further determine if changes at the transcriptional levels of efflux pumps could contribute to the altered antibiotic resistance phenotype in the *Δhns* mutant background*,* the transcriptomic data of AB5075 *Δhns* vs. AB5075 was examined. Our results showed that the expression of genes encoding AdeABC, AdeIJK as well as the efflux pump EmrAB were enhanced in AB5075 *Δhns*; however, the expression of genes that encode for AdeFGH were decreased (Fig. 2A and Supplementary Table S2). qRT-PCR experiments further confirmed up-regulation of efflux pump genes in both *A. baumannii Δhns* mutant strains when compared with otherwise isogenic wild-type strains (Fig. 2B and Supplementary S3). *In trans* complementation restored the expression levels for these genes in the AB5075 strain. The up-regulation of efflux pumps can be related with the wide regulatory effects of H-NS causing transcriptional changes in genes related with *A. baumannii*’s adaptative response.

We observed a wide transcriptional response in the *Δhns* mutant, pointing out the central role of H-NS in modulating expression/repression of a wide variety of antibiotic resistance genes. Our results confirmed a pleiotropic role of H-NS as a transcriptional regulator that is playing a critical role in reprogramming the *A. baumannii* transcriptome related with antibiotic resistance and stress response.

### Biofilm and Quorum sensing networks are also affected by H-NS

**Figure 3:**
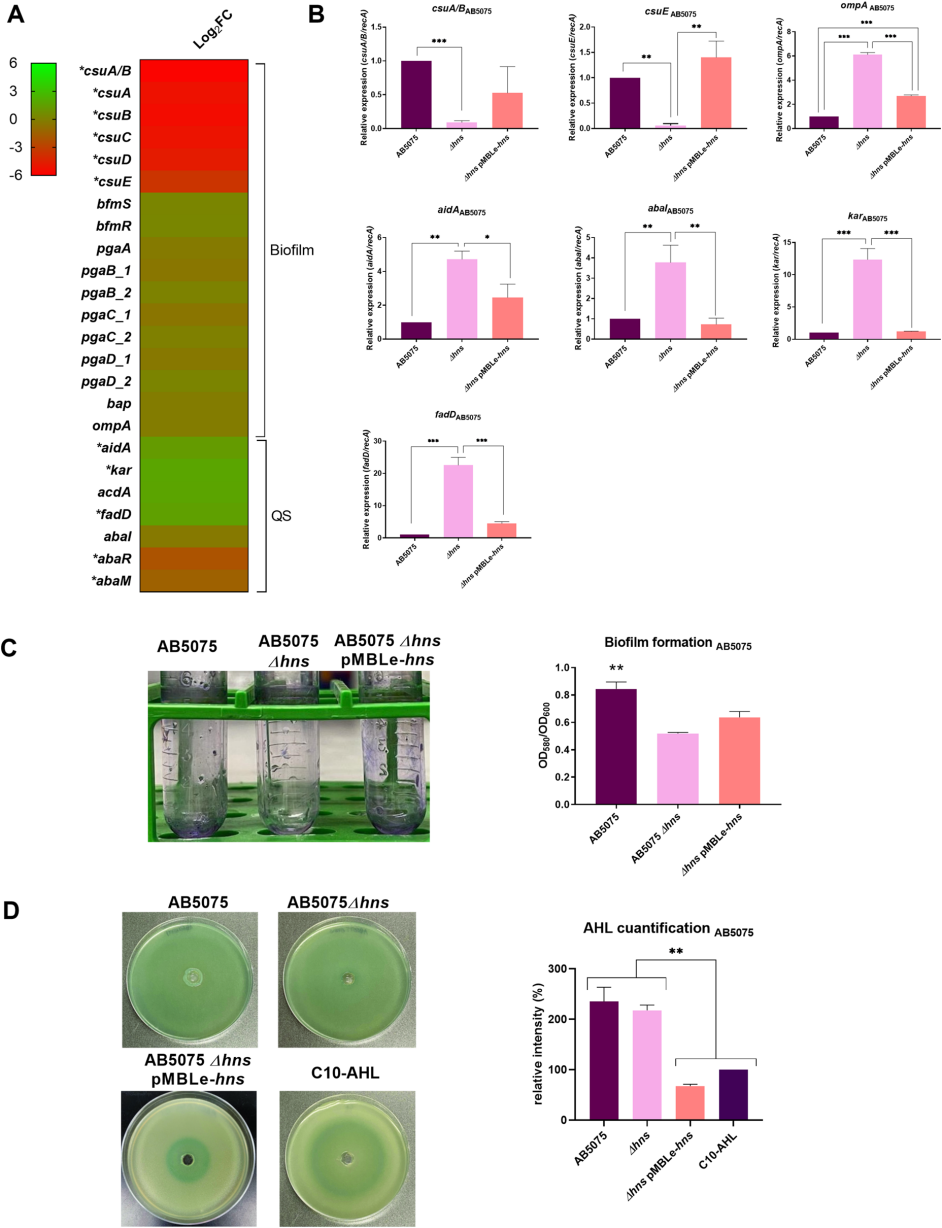
Genetic and phenotypic analysis of biofilm and quorum sensing coding genes. (**A**) Heatmap outlining the differential expression of genes associated with biofilm formation and quorum sensing in *A. baumannii* AB5075 *Δhns* vs. parental strain. The asterisks represent the DEGs (adjusted *P*-value < 0.05 with log2fold change > 1). Heat map was performed using GraphPad Prism version number 9 (GraphPad software, San Diego, CA, USA, https://www.graphpad.com/) (**B**) qRT-PCR of AB5075, AB5075 *Δhns* and AB5075 *Δhns* pMBLe-*hns* strains genes associated with biofilm and quorum sensing, the expression levels and the fold changes were determinate using the double ΔCt analysis. At least three independent samples were used, and three technical replicates were performed from each sample. Statistical significance (*P*-value < 0.05) was determined by ANOVA followed by Tukey’s multiple comparison test; one asterisks: *P-*value < 0.05; two asterisks: *P*-value < 0.01 and three asterisks: *P*-value < 0.001. (**C**) Biofilm assays performed in *A. baumannii* AB5075, AB5075 *Δhns* and AB5075 *Δhns* pMBLe-*hns* represented by OD580/OD600. Statistical analysis (*t* test) was performed and a *P-value* < 0.05 was considered significant. (**D**) Agar plate assay for the detection of AHL using *A. tumefaciens.* The presence of AHL was determined by the development of the blue color. Quantification of 5,5′-dibromo-4,4′-dichloro-indigo were estimated as the percentage relative to C10-AHL standard, measured with ImageJ (NIH). The mean ± SD is informed. Statistical significance (*P*-value < 0.05) was determined by ANOVA followed by Tukey’s multiple-comparison test. Experiments were performed in triplicate, with at least three technical replicates per biological replicate. This figure was performed using GraphPad Prism version number 9 (GraphPad software, San Diego, CA, USA, https://www.graphpad.com/).

The transcriptomic comparison of *A. baumannii* AB5075 Δ*hns* against the parental strain showed that expression of *csu* operon, which is essential for *A. baumannii* biofilm formation^30^, was significantly down-regulated (Fig. 3A and Supplementary Table S2). The differential expression of *csuA/B* and *csuE* was further confirmed to be down-regulated by qRT-PCR analysis in AB5075 *Δhns* (Fig. 3B), while the AB5075 *Δhns* pMBLe-*hns* restored the wild-type level of expression of both *csu* genes analyzed. In addition, the changes in expression of these genes were correlated with the associated phenotype, since biofilm formation was significantly lower in AB5075 *Δhns* with respect to the parental strain (Fig. 3C). The rescued strain showed a slightly increment of biofilm formation compared to the *Δhns* strain, however, statistics differences were not observed between mutant and complementary strain (Fig. 3C). Decreased biofilm formation was also seen in A118 *Δhns* and a decreased in the expression level of *csuE* was also observed in the mutant background (Supplementary Fig. S4A,B). Levels of expression of *ompA*, that encodes for the OmpA protein, partially involved in abiotic biofilm formation and known to be a virulence factor^31^, were up-regulated in both mutants strains (Fig. 3B and Supplementary Fig. S4A).

It is known that in a biofilm the microorganisms reside and communicate with each other through quorum sensing mechanisms^32^. AB5075 transcriptomic data analysis revealed an upregulation of the lactonase enzyme coding *aidA* gene and genes encoding enzymes that participate in acyl homoserine lactone (AHL) synthesis (*kar, acdA, fadD, abaI, abaR* and *abaM*)^33,34^ in the mutant strain compared to wild type strain (Fig. 3A and Supplementary Table S2). The quorum network genes *aidA, abaI, kar*, and *fadD* were confirmed to be up-regulated in AB5075 *Δhns* mutant strains by qRT-PCR and the levels of expression were restored in *hns* complemented strain (Fig. 3B). Moreover, the levels of expression of *aidA* and *abaI* genes were observed to be up-regulated in the susceptible model strain A118 (Supplementary Fig. S4A). In addition, we assessed the levels of AHL using *Agrobacterium tumefaciens*-based solid plate assays^35,36^. The supernatants from cultures of AB5075 or AB5075 *Δhns* produced similar intensity of color, likely caused by an increase of both lactonase activity (quorum quenching) and AHL synthesis (quorum sensing) in the mutant strain (Fig. 3D). The lower AHL production observed in the AB5075 *Δhns* pMBLe-*hns* can be explained by the decrease expression of genes associated with AHL synthesis (*abaI, karD,* and *fadD*) and the higher lactonase expression levels responsible of quorum quenching activity (*aidA*), therefore in this strain the degradation of AHL is more evident. Finally, AHL analysis for A118 *Δhns* showed lower intensity of blue color compared to A118 wild type strain (Supplementary Fig. S4C). This result suggested the production of a lactonase activity that is able to counteract the levels of large chain AHLs synthesized by this strain in the assayed conditions.

The genome-wide role of H-NS in *A. baumannii* was first studied in a hypermotile derivative of the reference strain ATCC 17978^7^. Transcriptomic analysis of the ATCC 17978 *Δhns* (strain 17978hm) identified 152 genes as DEGs (91 genes up-regulated and 61 down- regulated) when compared to the parental. The most striking up-regulation was observed in the quorum sensing genes encoding AbaI and the regulator AbaR. On the other hand, phenotypic and genetic analysis of biofilm coding genes showed no major differences between the wild type and mutant strains^7^. Our transcriptomic and phenotypic data compared to that of ATCC 17978, demonstrated that H-NS regulon components might vary in a strain-specific manner consistent with the fact that different *A. baumannii* isolates can display significant variations in phenotypes known to be controlled by H-NS (i.e., cell surface hydrophobicity, adherence and motility)^37^.

## Conclusion

Our present work further contributes to deciphering the role of the H-NS global regulator in the control of transcription of antibiotic resistance-related mechanisms.Using distinct *A. baumannii* model strains, an extensive H-NS-controlled transcriptional response was observed. Our results reinforce the complexity of the regulatory network control by the transcriptional repressor H-NS that regulates the expression of genes involved in antibiotic resistance and persistence. Overall, H-NS exerts a pleiotropic role in *A. baumannii*, influencing the expression of a wide variety of genes related to antibiotic resistance and stress response, suggesting that it is a good candidate to develop further alternative treatment approaches to combat *A. baumannii* infections.

## Materials and methods

### Bacterial strains

The multidrug and hypervirulent AB5075 strain and its isogenic *h-ns* mutant (AB5075 Δ*hns*) were used in the present study. AB5075 Δ*hns* was obtained from the *A. baumannii* AB5075 transposon mutant library^38^. In addition, to extend the role of H-NS on the response of *A. baumannii*, the model susceptible A118 and its isogenic mutant A118 *Δhns* were used. *A. baumannii* A118 Δ*hns* was obtained as previously described^39^*.* Briefly, chromosomal DNA of AB5075 Δ*hns* (*hns*::TET^R^) was used to naturally transform the A118 strain and mutant cells were selected on Luria–Bertani (LB) agar plates supplemented with 30 µg/mL tetracycline (TET). A118 Δ*hns* mutant strain was confirmed by automated DNA sequencing. Growth curves confirmed no differences in growth between the mutant and the complemented strains respect to the parental strains (Supplementary Fig. S5).

### Electroporation

Electro-competent *A. baumannii* AB5075 Δ*hns* cells were prepared and mixed with 25 ng of pMBLe-*hns* plasmid DNA (containing apramycin resistance) followed by electroporation with a Bio-Rad Gene Pulser instrument at 2.5 kV, 200 Ω, 25 µF. The electroporated cells were placed in recovery with 1 ml of LB broth for 2 h at 37 °C in a shaking incubator followed by culturing overnight at 37 °C on LB agar containing 15 µg/ml apramycin. At least 10 colonies were picked to confirm the presence of the different plasmids. To confirm their presence, plasmid extraction followed by gel electrophoresis analysis.

### RNA extraction and RNA-seq analysis

*A. baumannii* AB5075 and AB5075 Δ*hns* cells were cultured in LB broth and incubated with agitation for 18 h at 37 °C. Overnight cultures were then diluted 1:10 in fresh LB broth and incubated with agitation for 7 h at 37 °C. The Direct-zol RNA Kit (Zymo Research, Irvine, CA, USA) was used to perform the RNA extraction in triplicates. RNA samples were DNase treated (Thermo Fisher Scientific, Waltham, MA, USA) following manufacturer’s instruction. Samples were confirmed to have no DNA contamination through PCR amplification of the 16S rDNA gene. RNA sequencing was outsourced to Novogene (Novogene Corporation, Sacramento, CA, USA). Ribosomal RNA-depletion was done using the Ribo-Zero kit (Illumina) and the construction of the cDNA library was performed with the TruSeq Stranded Total RNA Library Prep kit (Illumina) from three independent replicates per sample. Analysis of the quality of the illumina reads, trimming of low-quality bases and removal of Illumina adapters was performed as described previously^17^. Reads were aligned to the genome of *A. baumannii* AB5075 using Burrows-Wheeler Alignment (BWA) software (v0.7.17) BWA and visualized using the Integrative Genomics Viewer (IGV). Read counts per gene were calculated using FeatureCounts^40^, and differential expression analysis was performed using DEseq2. Differentially expressed genes (DEGs) were defined as those displaying an FDR adjusted *P-value* of < 0.05 and log2fold change > 1. RNA-seq data generated as a result of this work has been deposited to SRA with the accession GSE167117.

qRT-PCR was performed to confirm and analyze the expression with antibiotic associated genes. cDNA was prepared using the iScript Reverse Transcription Supermix for qRT-PCR (BioRad, Hercules, CA, USA) and quantitative PCR was performed using iQ™SYBR Green Supermix (BioRad, Hercules, CA, USA) per the manufacturer’s recommendations, respectively. Results were analyzed using the 2^−ΔΔCt^ method in which *recA*^13^ acted as the control gene. Experiments were performed in technical and biological triplicates. The results from experiments performed were statistical analyzed (t test) using GraphPad Prism (GraphPad software, San Diego, CA, USA). A *P-*value < 0.05 was considered significant.

### Antimicrobial susceptibility assay

Antimicrobial commercial disks (BBL, Cockeysville, MD, USA) containing 10 μg of meropenem (MEM), 10 μg of ampicillin (AMP), 30 μg cefotaxime (CTX), 30 μg of ceftazidime (CAZ), 10 μg of imipenem (IMP), 15 μg of tigecycline (TIG), 30 μg of minocycline (MIN), 5 μg of ciprofloxacin (CIP), 10 μg of norfloxacin (NOR), 300 units of polymyxin B (PB), 30 μg of amikacin (AMK) and 10 μg of gentamicin (GN) were used. The inoculated plates were incubated at 37 °C for 18 h. MICs against were performed using the reference methods agar dilution according to CLSI Standards^41^. Antimicrobial commercial E-strips (Liofilchem S.r.l., Italy) for meropenem (MEM), ampicillin (AMP), ceftazidime (CAZ), imipenem (IMP), ciprofloxacin (CIP), norfloxacin (NOR), amikacin (AN), and gentamicin (GN) were used. Mueller–Hinton agar plates were incubated at 37 °C for 18 h. CLSI breakpoints were used for interpretation^41^. The MIC for colistin susceptibility testing was determined using ComASP Kit(Liofilchem) and following the manufacturer’s instructions.

### Biofilm assays

Biofilms assays were performed as described in previous work^13^. *A. baumannii* cells were cultured in LB broth with agitation for 18 h at 37 °C. Overnight cultures were centrifuged at 5000 rpm at 4 °C for 5 min. Pelleted cells were washed twice with 1X phosphate buffered saline (PBS) and then re-suspended in 1X PBS. The optical density at 600 nm (OD600) of each culture was then adjusted to 0.9–1.1 and the samples were the vortexed and diluted 1:100 in LB broth in technical triplicates, the inoculated 96-well polystyrene micro-titer platewas incubated at 37 °C for 24 h without agitation. The following day, the OD600 (ODG) was measured using a micro-plate reader (SpectraMaxM3 microplate/ cuvette reader with SoftMax Pro v6 software) to determine the total biomass. Wells were emptied using a vacuum pipette, washed three times with 1X PBS and stained with 1% crystal violet (CV) for 15 min. Excess CV was removed by washing three more times with 1X PBS and the biofilm associated with the CV was solubilized in ethanol acetate (80:20) for 30 m. The OD580 (ODB) was measured using a micro-plate reader and results were reported as the ratio of biofilm to total biomass (ODB/ODG). Experiments were performed in triplicates, with at least three technical replicates per biological replicate.

### *N*-acyl homoserine lactone (AHL) detection

*Agrobacterium tumefaciens*-based solid plate assays were carried out to detect N-Acyl Homoserine Lactone (AHL) production^42^ as described in previous work^36^. Initially, 500 µL of the homogenate were loaded in a central well of 0.7% LB agar plates supplemented with 40 µg of 5-bromo-3-indolyl-b-d-galactopyranoside (X-Gal) per mL and 250 µL (OD = 2.5) of the overnight culture of *Agrobacterium tumefaciens* biosensor. The presence of AHL was determined by the development of blue coloring^35^. As a positive control, 100 µL of *N*-Decanoyl-dl-homoserine lactone (C10-AHL) 12.5 mg/mL was utilized. Quantification of 5,5′-dibromo-4,4′-dichloro-indigo production in different conditions was determined using ImageJ software (NIH) by measuring the intensity of each complete plate, and subtracting the intensity measured in the negative control. The values were normalized to the positive control, which received the arbitrary value of 100.

### Growth of *A. baumannii* strains

To test the ability of the *A. baumannii* A118 and AB5075 wild-type and derivative strains used in this work, 1/100 dilutions of overnight cultures grown in LB were inoculated in LB. Cultures were subsequently grown in agitation at 37 °C. Optical density of 600 nm was measured using a micro-plate reader (SpectraMax M3 microplate/cuvette reader with SoftMax Pro v6 software).

### Statistical analysis

Experiments performed at least in triplicates were statistically analyzed by ANOVA followed by Tukey’s multiple comparison test using GraphPad Prism (GraphPad software, San Diego, CA, USA). A *P-*value < 0.05 was considered significant.

All procedures performed in this study were in accordance with the CSUF Institutional Biosafety Committee Approval plan (DBH117-01) and are in compliance with the NIH, CDC, OSHA and other environmental and occupational regulations.

## Supplementary Information

Supplementary Information.
