# Supplementary material for: Histone-like nucleoid-structuring protein (H-NS) regulatory role in antibiotic resistance in Acinetobacter baumannii
Source: Sci Rep. 2021 Sep 16;11:18414. doi: 10.1038/s41598-021-98101-w (PMC8446060; doi:10.1038/s41598-021-98101-w)
Supplement: Supplementary file 1 — Supplementary Information. [file 41598_2021_98101_MOESM1_ESM.docx]

**Additional Information**

**Supplementary Table S1:**

| **Gene ID** | **log2FoldChange** | **FDR (*P-*adjusted)** | **Gene Function** |
| --- | --- | --- | --- |
| A591_A0067 | -1,2952 | 2,02E-06 | "putative N-acetyltransferase YedL" |
| A591_A0102 | -2,5581 | 1,61E-27 | "PF07007 family protein" |
| A591_A0103 | -3,4432 | 1,56E-48 | "SH3 domain protein" |
| A591_A0277 | 2,0968 | 5,21E-12 | "prepilin-type cleavage/methylation N-terminal |
| A591_A0280 | -1,1748 | 6,11E-05 | "putative bacterioferritin-associated ferredoxin" |
| A591_A0309 | 1,8422 | 9,79E-25 | "transporter, major facilitator family protein" |
| A591_A0321 | -1,4806 | 2,96E-42 | "gdhA, glutamate dehydrogenase" |
| A591_A0322 | -1,3758 | 2,13E-37 | "astC, succinylornithine transaminase family protein" |
| A591_A0323 | -1,3164 | 1,16E-25 | "astA_1, arginine N-succinyltransferase" |
| A591_A0324 | -1,1573 | 5,45E-15 | "astD, succinylglutamate-semialdehyde dehydrogenase" |
| A591_A0325 | -1,1162 | 5,68E-13 | "astB, succinylarginine dihydrolase" |
| A591_A0326 | -1,1559 | 3,46E-12 | "astE, succinylglutamate desuccinylase" |
| A591_A0345 | 1,7022 | 8,56E-05 | "acyl-CoA dehydrogenase N-terminal domain protein" |
| A591_A0558 | 2,2536 | 2,13E-07 | "PF11776 domain protein" |
| A591_A0637 | 1,0755 | 6,18E-09 | "pilJ, protein PilJ" |
| A591_A0669 | -1,0200 | 4,43E-05 | "putative N-acetyltransferase YedL" |
| A591_A0738 | 1,0673 | 2,36E-13 | "PF14076 domain protein" |
| A591_A0868 | 1,2675 | 3,38E-08 | "transglycosylase SLT domain protein" |
| A591_A0872 | -3,2128 | 5,96E-45 | "putative N-acetyltransferase YedL" |
| A591_A0873 | -1,2753 | 2,06E-04 | "putative N-acetyltransferase YedL" |
| A591_A0874 | -1,3430 | 2,80E-02 | "putative N-acetyltransferase YedL" |
| A591_A0875 | -1,0121 | 5,18E-07 | "putative N-acetyltransferase YedL" |
| A591_A1096 | -1,6689 | 2,77E-07 | "putative inner membrane protein YddG" |
| A591_A1097 | -1,1854 | 9,43E-05 | "EamA-like transporter family protein" |
| A591_A1098 | -2,5088 | 3,89E-11 | "putative prevent host death protein" |
| A591_A1099 | -3,6893 | 3,56E-172 | "transcriptional regulator, TetR family" |
| A591_A1101 | -2,0145 | 3,45E-23 | "putative N-acetyltransferase YedL" |
| A591_A1110 | 1,5589 | 4,66E-08 | "condensation domain protein" |
| A591_A1114 | 1,1574 | 4,54E-03 | "ABC transporter, ATP-binding protein" |
| A591_A1192 | -1,3297 | 1,53E-02 | "putative N-acetyltransferase YedL" |
| A591_A1200 | -1,2614 | 4,79E-02 | "putative N-acetyltransferase YedL" |
| A591_A1209 | -1,1815 | 3,90E-02 | "putative N-acetyltransferase YedL" |
| A591_A1338 | -1,0788 | 3,57E-02 | "putative N-acetyltransferase YedL" |
| A591_A1360 | -3,1669 | 5,95E-39 | "acyltransferase" |
| A591_A1391 | -2,1747 | 2,19E-12 | "putative N-acetyltransferase YedL" |
| A591_A1394 | 1,5965 | 1,50E-03 | "phaC, poly(R)-hydroxyalkanoic acid synthase, class III, |
| A591_A1404 | -4,7721 | 2,10E-43 | "transcriptional regulator, TetR family" |
| A591_A1405 | -5,8326 | 4,23E-54 | "spore coat protein, U domain family" |
| A591_A1406 | -5,2083 | 1,13E-27 | "spore coat protein, U domain family" |
| A591_A1407 | -5,4607 | 8,86E-45 | "spore coat protein, U domain family" |
| A591_A1408 | -5,1481 | 1,43E-48 | "PapD pilus/flagellar-assembly chaperone |
| A591_A1409 | -4,7479 | 8,35E-94 | "chaperone-usher secretion system usher protein" |
| A591_A1410 | -3,5634 | 1,06E-33 | "csuE, protein CsuE" |
| A591_A1412 | -1,1011 | 2,95E-03 | "putative N-acetyltransferase YedL" |
| A591_A1481 | 1,6969 | 3,46E-29 | "luciferase family oxidoreductase, group 1" |
| A591_A1543 | -1,1469 | 9,24E-04 | "alcohol dehydrogenase, iron-dependent" |
| A591_A1559 | -1,0955 | 1,79E-02 | "putative N-acetyltransferase YedL" |
| A591_A1569 | -1,4409 | 1,47E-12 | "putative N-acetyltransferase YedL" |
| A591_A1576 | -1,5765 | 5,93E-04 | "putative N-acetyltransferase YedL" |
| A591_A1577 | -1,2043 | 3,19E-04 | "putative N-acetyltransferase YedL" |
| A591_A1589 | -1,4094 | 2,01E-06 | "transmembrane pair family protein" |
| A591_A1591 | 1,9695 | 7,68E-05 | "acetyl-CoA C-acyltransferase" |
| A591_A1592 | 1,7278 | 3,07E-05 | "KR domain protein" |
| A591_A1610 | -1,3553 | 1,32E-20 | "transcriptional regulator, TetR family" |
| A591_A1677 | -1,4046 | 3,09E-12 | "putative extracellular serine proteinase" |
| A591_A1691 | 1,0408 | 1,62E-04 | "putative lysophospholipase" |
| A591_A1773 | -2,0483 | 2,07E-04 | "putative glutamyl-tRNA(Gln) amidotransferase |
| A591_A1774 | -1,2821 | 1,62E-02 | "putative acyl-CoA dehydrogenase" |
| A591_A1775 | -1,6064 | 2,30E-03 | "putative N-acetyltransferase YedL" |
| A591_A1818 | -3,0152 | 6,76E-87 | "L-sorbosone dehydrogenase" |
| A591_A1819 | -3,3095 | 2,43E-71 | "putative N-acetyltransferase YedL" |
| A591_A1825 | -2,5175 | 2,40E-40 | "putative lipoprotein" |
| A591_A1830 | -1,4940 | 9,28E-12 | "putative membrane protein" |
| A591_A1831 | -1,0187 | 5,68E-13 | "putative N-acetyltransferase YedL" |
| A591_A1858 | 1,1408 | 6,87E-07 | "phosphoribosyl transferase domain protein" |
| A591_A1922 | 1,2187 | 7,37E-05 | "acetyltransferase (GNAT) domain protein" |
| A591_A1925 | 1,2720 | 1,55E-02 | "hypothetical protein" |
| A591_A1926 | 2,7175 | 7,86E-56 | "hypothetical protein" |
| A591_A1927 | 2,7663 | 3,69E-71 | "putative N-acetyltransferase YedL" |
| A591_A1928 | 1,0327 | 1,59E-09 | "putative N-acetyltransferase YedL" |
| A591_A1932 | -1,0127 | 4,24E-06 | "putative lipoprotein" |
| A591_A1933 | -1,5157 | 7,91E-04 | "putative N-acetyltransferase YedL" |
| A591_A1934 | -2,0336 | 1,35E-43 | "putative N-acetyltransferase YedL" |
| A591_A1935 | -2,3413 | 1,28E-19 | "putative N-acetyltransferase YedL" |
| A591_A1936 | -2,5744 | 3,10E-73 | "putative N-acetyltransferase YedL" |
| A591_A1938 | 1,6916 | 3,19E-25 | "toxic anion resistance protein TelA" |
| A591_A1939 | 1,1574 | 1,53E-09 | "putative N-acetyltransferase YedL" |
| A591_A1940 | 1,4259 | 1,42E-34 | "putative N-acetyltransferase YedL" |
| A591_A1941 | 1,1081 | 1,77E-19 | "ParB-like protein" |
| A591_A1942 | 1,4930 | 3,06E-30 | "CobQ/CobB/MinD/ParA nucleotide binding domain |
| A591_A1943 | 2,1162 | 1,69E-27 | "putative N-acetyltransferase YedL" |
| A591_A1944 | 2,8732 | 2,37E-26 | "putative N-acetyltransferase YedL" |
| A591_A1945 | 2,8636 | 2,33E-29 | "putative N-acetyltransferase YedL" |
| A591_A1983 | 1,5322 | 4,33E-04 | "putative type IV conjugative transfer system |
| A591_A1984 | 1,3625 | 8,57E-06 | "traL, type IV conjugative transfer system protein TraL" |
| A591_A1985 | 1,0979 | 1,62E-03 | "putative N-acetyltransferase YedL" |
| A591_A1987 | 1,9463 | 2,48E-07 | "transglycosylase SLT domain protein" |
| A591_A1996 | -1,3740 | 3,28E-05 | "putative N-acetyltransferase YedL" |
| A591_A1997 | -1,6145 | 7,04E-03 | "putative N-acetyltransferase YedL" |
| A591_A1998 | -3,8700 | 3,37E-27 | "putative N-acetyltransferase YedL" |
| A591_A1999 | -3,2336 | 2,37E-68 | "putative N-acetyltransferase YedL" |
| A591_A2005 | 2,4036 | 1,23E-37 | "putative N-acetyltransferase YedL" |
| A591_A2006 | 2,1402 | 1,19E-14 | "putative N-acetyltransferase YedL" |
| A591_A2007 | 1,2973 | 1,10E-06 | "putative N-acetyltransferase YedL" |
| A591_A2008 | 1,5442 | 4,92E-06 | "DnaJ domain protein" |
| A591_A2009 | 1,3796 | 4,55E-03 | "putative N-acetyltransferase YedL" |
| A591_A2022 | -1,0206 | 1,49E-20 | "catalase domain protein" |
| A591_A2034 | -1,2080 | 3,08E-02 | "pqqA, coenzyme PQQ biosynthesis protein A" |
| A591_A2049 | -1,0419 | 4,22E-02 | "flavin reductase-like protein" |
| A591_A2055 | -1,5899 | 4,22E-21 | "3-hydroxybutyrate dehydrogenase" |
| A591_A2056 | -2,0385 | 2,13E-37 | "citrate transporter" |
| A591_A2090 | -1,3524 | 3,36E-07 | "budC, diacetyl reductase ((S)-acetoin forming)" |
| A591_A2091 | -1,5027 | 6,70E-15 | "lpdA_2, dihydrolipoyl dehydrogenase" |
| A591_A2092 | -1,9178 | 8,61E-15 | "putative dihydrolipoyllysine-residue |
| A591_A2093 | -2,0040 | 2,75E-13 | "acoB, acetoin:2,6-dichlorophenolindophenol |
| A591_A2094 | -2,6477 | 1,04E-32 | "acoA, acetoin:2,6-dichlorophenolindophenol |
| A591_A2095 | -3,7998 | 7,30E-105 | "lipA_2, lipoyl synthase" |
| A591_A2115 | 1,2708 | 4,56E-04 | "alpha/beta hydrolase family protein" |
| A591_A2272 | -1,6680 | 8,65E-63 | "fimbrial protein" |
| A591_A2273 | -1,9891 | 2,43E-37 | "gram-negative pili assembly chaperone protein" |
| A591_A2274 | -1,3045 | 7,87E-23 | "chaperone-usher secretion system usher protein" |
| A591_A2284 | -1,0350 | 3,26E-08 | "site-specific recombinase, phage integrase |
| A591_A2286 | -2,3902 | 7,98E-56 | "putative N-acetyltransferase YedL" |
| A591_A2287 | -2,8740 | 8,79E-06 | "transcriptional regulator, TetR family" |
| A591_A2288 | -1,1514 | 2,45E-14 | "acyltransferase" |
| A591_A2321 | -2,2222 | 2,87E-106 | "ansB, glutamin-(asparagin-)ase" |
| A591_A2344 | 1,0115 | 2,15E-07 | "NMT1/THI5-like protein" |
| A591_A2347 | -2,9207 | 4,61E-46 | "FMN-dependent oxidoreductase, nitrilotriacetate |
| A591_A2350 | 1,2401 | 9,83E-05 | "cytochrome ubiquinol oxidase" |
| A591_A2378 | -1,1832 | 2,89E-04 | "major membrane protein 1 family protein" |
| A591_A2396 | -1,7630 | 1,00E-02 | "putative N-acetyltransferase YedL" |
| A591_A2397 | -2,0152 | 8,83E-06 | "putative N-acetyltransferase YedL" |
| A591_A2399 | -1,1213 | 4,97E-05 | "putative N-acetyltransferase YedL" |
| A591_A2400 | -1,3153 | 2,89E-11 | "Iron-containing redox enzyme" |
| A591_A2401 | -1,3602 | 1,22E-07 | "cinA_1, competence/damage-inducible protein CinA" |
| A591_A2403 | -1,4327 | 4,94E-04 | "putative N-acetyltransferase YedL" |
| A591_A2471 | -1,2837 | 2,07E-02 | "putative N-acetyltransferase YedL" |
| A591_A2472 | -2,5158 | 3,77E-13 | "putative N-acetyltransferase YedL" |
| A591_A2473 | -3,4688 | 2,35E-32 | "putative N-acetyltransferase YedL" |
| A591_A2475 | -3,7855 | 1,41E-49 | "putative N-acetyltransferase YedL" |
| A591_A2476 | -4,1323 | 5,75E-44 | "hypothetical protein" |
| A591_A2477 | -6,4098 | 6,78E-82 | "cspG_2, cold shock-like protein CspG" |
| A591_A2478 | -2,2641 | 9,30E-04 | "putative N-acetyltransferase YedL" |
| A591_A2479 | -2,6744 | 9,13E-04 | "putative N-acetyltransferase YedL" |
| A591_A2480 | -1,4043 | 2,76E-10 | "putative N-acetyltransferase YedL" |
| A591_A2514 | -1,4517 | 9,47E-13 | "soxR, redox-sensitive transcriptional activator SoxR" |
| A591_A2515 | -1,5238 | 3,60E-20 | "putative N-acetyltransferase YedL" |
| A591_A2567 | -1,3825 | 1,67E-13 | "putative N-acetyltransferase YedL" |
| A591_A2568 | -2,0614 | 3,10E-36 | "putative N-acetyltransferase YedL" |
| A591_A2583 | -4,3539 | 0,00E+00 | "putative lipoprotein" |
| A591_A2613 | -1,8870 | 9,01E-59 | "putative N-acetyltransferase YedL" |
| A591_A2633 | -1,1511 | 5,41E-04 | "putative N-acetyltransferase YedL" |
| A591_A2638 | -4,1281 | 3,50E-135 | "glycine zipper" |
| A591_A2639 | -1,8623 | 9,27E-06 | "PF13628 domain protein" |
| A591_A2647 | -1,4885 | 3,90E-34 | "putative pyrroline-5-carboxylate reductase" |
| A591_A2648 | -1,6878 | 3,24E-06 | "putative N-acetyltransferase YedL" |
| A591_A2649 | -1,0328 | 4,92E-16 | "cspE, cold shock-like protein CspE" |
| A591_A2671 | -1,3145 | 7,95E-03 | "MFS transporter, aromatic acid:H+ symporter |
| A591_A2672 | -1,1821 | 1,26E-03 | "outer membrane porin, OprD family" |
| A591_A2753 | -3,3509 | 1,04E-66 | "putative N-acetyltransferase YedL" |
| A591_A2754 | -2,8246 | 2,59E-42 | "PF14085 domain protein" |
| A591_A2755 | -2,8036 | 3,08E-142 | "putative N-acetyltransferase YedL" |
| A591_A2768 | -1,7198 | 9,09E-123 | "AAA domain protein" |
| A591_A2769 | -2,3951 | 7,10E-41 | "putative lipoprotein" |
| A591_A2788 | -1,0683 | 1,28E-06 | "putative N-acetyltransferase YedL" |
| A591_A2883 | -1,0548 | 1,91E-02 | "transporter, major facilitator family protein" |
| A591_A2918 | -1,3412 | 1,68E-03 | "putative N-acetyltransferase YedL" |
| A591_A2962 | 2,0073 | 3,43E-05 | "putative N-acetyltransferase YedL" |
| A591_A2963 | 2,5455 | 1,08E-07 | "BRO family, N-terminal domain protein" |
| A591_A2964 | 2,7214 | 1,21E-04 | "DNA-binding helix-turn-helix protein" |
| A591_A2965 | 2,7762 | 3,78E-04 | "putative N-acetyltransferase YedL" |
| A591_A3065 | -1,2972 | 6,50E-22 | "BioH family protein" |
| A591_A3066 | -1,1742 | 3,56E-06 | "otsB, trehalose-phosphatase" |
| A591_A3128 | -2,2112 | 1,61E-12 | "putative N-acetyltransferase YedL" |
| A591_A3132 | -2,7383 | 3,13E-45 | "putative N-acetyltransferase YedL" |
| A591_A3133 | -2,1332 | 9,38E-32 | "putative N-acetyltransferase YedL" |
| A591_A3134 | -1,1880 | 3,38E-08 | "putative N-acetyltransferase YedL" |
| A591_A3283 | -1,1448 | 9,90E-07 | "transcriptional regulator, TetR family" |
| A591_A3412 | 1,5231 | 3,45E-07 | "AMP-binding enzyme" |
| A591_A3434 | -1,1915 | 4,49E-03 | "putative N-acetyltransferase YedL" |
| A591_A3532 | -2,6368 | 2,17E-42 | "H-NS histone family protein" |
| A591_A3601 | 1,1093 | 3,95E-02 | "MCM2/3/5 family protein" |
| A591_A3685 | -1,3559 | 6,42E-08 | "MMPL family protein" |
| A591_A3686 | -1,5854 | 2,30E-11 | "AMP-binding enzyme" |
| A591_A3687 | -1,8778 | 3,24E-04 | "phosphopantetheine attachment domain protein" |
| A591_A3688 | -1,5903 | 2,93E-04 | "acyl-CoA dehydrogenase, C-terminal domain |
| A591_A3689 | -2,1116 | 1,82E-08 | "AMP-binding enzyme" |
| A591_A3690 | -2,1316 | 6,43E-26 | "putative autoinducer-binding transcriptional |
| A591_A3691 | -1,4556 | 2,21E-04 | "putative N-acetyltransferase YedL" |
| A591_A3712 | 1,2272 | 1,35E-05 | "oxidoreductase, short chain |
| A591_A3789 | 1,4779 | 4,67E-07 | "dual-action HEIGH metallo-peptidase" |
| A591_B0005 | 2,5700 | 7,79E-39 | "PF04365 family protein" |
| A591_B0006 | 2,0791 | 2,13E-34 | "PF14384 domain protein" |

**Supplementary Table S1:** Transcriptome analysis of *A. baumannii* strain AB5075 versus AB5075 *Δhns* showing the genes with an FDR-adjusted *P-value <* 0.05 and log2-fold change >1.

**Supplementary Table S2**

| **Gene ID** | **Gene Name** | **log2FoldChange** | **FDR (*P-*adjusted)** | **Gene Function** |  |  |  |  |
| --- | --- | --- | --- | --- | --- | --- | --- | --- |
| A591_A0079 | *advA* | 0,0464 | 6,80E-01 | "putative N-acetyltransferase YedL" | | |  |  |
| A591_A0081 | *aciT* | 0,2376 | 7,07E-02 | "putative N-acetyltransferase YedL" | | |  |  |
| A591_A0134 | *lpxC* | -0,1010 | 4,91E-01 | "lpxC, UDP-3-O-[3-hydroxymyristoyl] N-acetylglucosamine" | | | | |
| A591_A0256 | *pbp3* | 0,1010 | 2,78E-01 | "penicillin-binding protein, transpeptidase domain" | | | |  |
| A591_A0309 | *cmr* | 1,8422 | 9,79E-25 | "transporter, major facilitator family protein" | | | |  |
| A591_A0339 | *tetA_1* | -0,3349 | 3,40E-01 | "putative tetracycline resistance protein, class" | | | |  |
| A591_A0345 | *acdA* | 1,7022 | 8,56E-05 | "acyl-CoA dehydrogenase N-terminal domain protein" | | | |  |
| A591_A0407 | *sdhE* | -0,5502 | 3,79E-03 | "flavinator of succinate dehydrogenase" | | |  |  |
| A591_A0466 | *ompA* | -0,2046 | 1,89E-01 | "OmpA/MotB/Pal peptidoglycan-associating domain" | | | |  |
| A591_A0549 | *lpsB* | -0,5509 | 7,59E-08 | "LPS glycosyltransferase" | |  |  |  |
| A591_A0786 | *adeK* | 0,4618 | 4,49E-03 | "putative outer membrane efflux protein OprM" | | | |  |
| A591_A0787 | *adeJ* | 0,5201 | 2,21E-04 | "mexB, multidrug resistance protein MexB" | | |  |  |
| A591_A0788 | *adeI* | 0,5330 | 3,71E-04 | "putative acriflavine resistance protein A" | | |  |  |
| A591_A0813 | *sdhC* | 0,2112 | 3,32E-01 | "sdhC, succinate dehydrogenase, cytochrome b556 subunit" | | | | |
| A591_A0814 | *sdhD* | 0,2114 | 1,16E-01 | "sdhD, succinate dehydrogenase, hydrophobic membrane" | | | | |
| A591_A0815 | *sdhA* | 0,3530 | 1,91E-02 | "sdhA, succinate dehydrogenase, flavoprotein subunit" | | | |  |
| A591_A0834 | *clmA* | 0,2474 | 3,77E-01 | "putative chloramphenicol O-acetyltransferase" | | | |  |
| A591_A0856 | *bap* | -0,2000 | 1,33E-01 | "type I secretion C-terminal target domain | | |  |  |
| A591_A0954 | *carO* | 0,3356 | 6,22E-02 | "beta-lactamase" |  |  |  |  |
| A591_A1009 | *bla*GES-14 | -0,2493 | 9,74E-02 | "class A beta-lactamase" | |  |  |  |
| A591_A1099 | TetR family | -3,6893 | 3,56E-172 | "transcriptional regulator, TetR family" | | |  |  |
| A591_A1133 | *bla*ADC | 0,0200 | 9,04E-01 | "beta-lactamase" |  |  |  |  |
| A591_A1260 | *adeH* | -0,2471 | 4,55E-01 | "oprC, outer membrane efflux protein OprC" | | | |  |
| A591_A1261 | *adeG* | -0,1051 | 8,41E-01 | "bpeF, multidrug efflux pump BpeF" | | |  |  |
| A591_A1262 | *adeF* | -0,1941 | 6,74E-01 | "bpeE, multidrug efflux pump BpeE" | | |  |  |
| A591_A1284 | *pbp1* | 0,1480 | 1,94E-01 | "mrcB, penicillin-binding protein 1B" | | |  |  |
| A591_A1386 | *dnpA* | -0,2739 | 4,36E-01 | "N-acetylglucosaminylphosphatidylinositol" | | |  |  |
| A591_A1404 | TetR family | -4,7721 | 2,10E-43 | "transcriptional regulator, TetR family" | | |  |  |
| A591_A1405 | *csuA/B* | -5,8326 | 4,23E-54 | "spore coat protein, U domain family" | | |  |  |
| A591_A1406 | *csuA* | -5,2083 | 1,13E-27 | "spore coat protein, U domain family" | | |  |  |
| A591_A1407 | *csuB* | -5,4607 | 8,87E-45 | "spore coat protein, U domain family" | | |  |  |
| A591_A1408 | *csuC* | -5,1481 | 1,43E-48 | "PapD pilus/flagellar-assembly chaperone" | | |  |  |
| A591_A1409 | *csuD* | -4,7479 | 8,35E-94 | "chaperone-usher secretion system usher protein" | | | |  |
| A591_A1410 | *csuE* | -3,5634 | 1,06E-33 | "csuE, protein CsuE" | |  |  |  |
| A591_A1454 | *prmA* | 0,1661 | 2,91E-01 | "prmA, ribosomal protein L11 methyltransferase" | | | |  |
| A591_A1476 | *pgaA* | -0,2708 | 9,00E-02 | "pgaA, poly-beta-1,6 N-acetyl-D-glucosamine export porin" | | | | |
| A591_A1477 | *pgaB_1* | -0,4640 | 3,69E-03 | "poly-beta-1,6-N-acetyl-D-glucosamine" | | |  |  |
| A591_A1478 | *pgaC_1* | -0,5225 | 3,84E-02 | "pgaC_1, poly-beta-1,6 N-acetyl-D-glucosamine synthase" | | | | |
| A591_A1479 | *pgaD_1* | -0,3698 | 1,51E-01 | "pgaD, poly-beta-1,6-N-acetyl-D-glucosamine biosynthesis" | | | | |
| A591_A1487 | *prmC* | 0,1437 | 5,11E-01 | "prmC, protein-(glutamine-N5) methyltransferase, release" | | | | |
| A591_A1592 | *kar* | 1,7278 | 3,07E-05 | "KR domain protein" | |  |  |  |
| A591_A1610 | *tetC* | -1,3553 | 1,32E-20 | "transcriptional regulator, TetR family" | | |  |  |
| A591_A1661 | *lpxD* | 0,0658 | 6,63E-01 | "lpxD, UDP-3-O-[3-hydroxymyristoyl] glucosamine" | | | |  |
| A591_A1663 | *lpxA* | -0,0520 | 8,18E-01 | "lpxA, acyl-[acyl-carrier-protein]-UDP-N-acetylglucosamine" | | | | |
| A591_A1888 | *adeS* | 0,2418 | 1,99E-01 | "putative N-acetyltransferase YedL" | | |  |  |
| A591_A1889 | *adeR* | -0,0806 | 7,22E-01 | "GHKL domain protein" | |  |  |  |
| A591_A1891 | *adeA* | 0,6665 | 1,22E-04 | "efflux transporter, RND family, MFP subunit" | | | |  |
| A591_A1892 | *adeB* | 0,7268 | 1,94E-08 | "RND transporter, HAE1 family" | | |  |  |
| A591_A1893 | *adeC* | 0,4494 | 2,53E-03 | "putative outer membrane efflux protein OprM" | | | |  |
| A591_A1902 | *aadA1* | 0,2229 | 8,02E-01 | "hypothetical protein" | |  |  |  |
| A591_A1912 | *aadB* | -0,2004 | 4,43E-01 | "putative gentamicin 2''-nucleotidyltransferase" | | | |  |
| A591_A1914 | *strA* | 0,9924 | 5,53E-05 | "streptomycin 3''-adenylyltransferase" | | |  |  |
| A591_A1915 | *strB* | 0,5241 | 2,32E-01 | "aphE, streptomycin 3''-kinase" | | |  |  |
| A591_A1916 | *aphA-6* | 0,8577 | 6,47E-02 | "aminoglycoside/hydroxyurea antibiotic resistance" | | | |  |
| A591_A1918 | *bla*OXA-23 | 0,6587 | 4,24E-06 | "beta-lactamase" |  |  |  |  |
| A591_A1919 | *aacA* | 0,5766 | 1,09E-04 | "aacA, aminoglycoside N(6')-acetyltransferas" | | | |  |
| A591_A1920 | *drfA7_1* | 0,3460 | 5,10E-04 | "folA_1, dihydrofolate reductase" | | |  |  |
| A591_A1921 | *sul1* | 0,9742 | 2,96E-12 | "folP_1, dihydropteroate synthase" | | |  |  |
| A591_A1922 | *aac(6')-Ib3* | 1,2187 | 7,37E-05 | "acetyltransferase (GNAT) domain protein" | | |  |  |
| A591_A2100 | *prmB* | 0,0705 | 6,16E-01 | "prmB, (glutamine-N5) methyltransferase, ribosomal" | | | |  |
| A591_A2115 | *aidA* | 1,2708 | 4,56E-04 | "alpha/beta hydrolase family protein" | | |  |  |
| A591_A2127 | *lpxB* | 0,1508 | 3,45E-01 | "lpxB, lipid-A-disaccharide synthase" | | |  |  |
| A591_A2262 | *bla*OXA51-like | -0,4435 | 4,90E-03 | "penicillin-binding protein, transpeptidase domain" | | | |  |
| A591_A2287 | TetR family | -2,8740 | 8,79E-06 | "transcriptional regulator, TetR family" | | |  |  |
| A591_A2830 | *pbp2* | 0,0835 | 5,89E-01 | "mrdA, penicillin-binding protein 2" | | |  |  |
| A591_A2911 | *pgaD_2* | 0,2056 | 2,91E-01 | "enoyl-CoA hydratase/isomerase family protein" | | | |  |
| A591_A2912 | *pgaC_2* | -0,0069 | 9,71E-01 | "pgaC_2, poly-beta-1,6 N-acetyl-D-glucosamine synthase" | | | | |
| A591_A2913 | *pgaB_2* | 0,0846 | 5,76E-01 | "pgaB, poly-beta-1,6-N-acetyl-D-glucosamine" | | | |  |
| A591_A2969 | *emrB* | 0,5988 | 4,33E-04 | "efflux pump membrane protein" | | |  |  |
| A591_A2970 | *emrA* | 0,2694 | 2,92E-01 | "drug resistance MFS transporter, drug:H+ | | |  |  |
| A591_A3011 | *fusA* | 0,5249 | 8,95E-04 | "fusA, translation elongation factor G" | | |  |  |
| A591_A3123 | *bfmS* | 0,2372 | 3,57E-02 | "GHKL domain protein" | |  |  |  |
| A591_A3124 | *bfmR* | 0,3004 | 1,60E-01 | "transcriptional regulatory protein, C-terminal" | | | |  |
| A591_A3283 | TetR family | -1,1448 | 9,90E-07 | "transcriptional regulator, TetR family" | | |  |  |
| A591_A3412 | *fadD* | 1,5231 | 3,45E-07 | "AMP-binding enzyme" | |  |  |  |
| A591_A3690 | *abaR* | -2,1316 | 6,43E-26 | "putative autoinducer-binding transcriptional" | | | |  |
| A591_A3691 | *abaM* | -1,4556 | 2,21E-04 | "putative N-acetyltransferase YedL" | | |  |  |
| A591_A3692 | *abaI* | -0,3455 | 6,30E-01 | "putative acyl-homoserine-lactone synthase" | | | |  |

**Supplementary Table S2:** Transcriptome analysis of *A. baumannii* strain AB5075 versus AB5075 *Δhns* showing the genes associated with antibiotic resistance, biofilm formation and quorum sensing.

**Supplementary Table S3**

|  | MEM | AMP | CTX | CAZ | IMP | TGC | MIN | CIP | NOR | PB | AN | GN |
| --- | --- | --- | --- | --- | --- | --- | --- | --- | --- | --- | --- | --- |
|  | Halo of inhibition (mm) | | | | | | | | | | | |
| AB5075 | 7 | 6 | 6 | 6 | 12 | 23 | 20 | 6 | 6 | 13 | 12 | 10 |
| AB5075 ∆*hns* | 12 | 6 | 6 | 6 | 16 | 26 | 22 | 6 | 6 | 15 | 16 | 19 |
| A118 | 26 | 10 | 20 | 22 | 30 | 22 | 28 | 30 | 21 | 15 | 24 | 22 |
| A118 ∆*hns* | 30 | 12 | 22 | 25 | 37 | 25 | 28 | 31 | 24 | 16 | 32 | 33 |

MEM: meropenem; AMP: ampicillin; CTX: cefotaxime; CAZ: ceftazidime; IMP: imipenem; TGC: tigecycline; MIN: minocycline; CIP: ciprofloxacin; NOR: norfloxacin; PB: polymyxin B; AN: amikacin; GN: gentamicin.

**Supplementary Table S3:** Disk-diffusion assays were performed and changes in the halo of inhibition were recorded for meropenem (MEM), ampicillin (AMP), cefotaxime (CTX), ceftazidime (CAZ), imipenem (IMP), tigecycline (TGC), minocycline (MIN), ciprofloxacin (CIP), norfloxacin (NOR), vancomycin (VAN), polymyxin B/colistin (PB/CST), amikacin (AN) and gentamicin (GN) in *A. baumannii* AB5075, AB5075 *Δhns*, A118 and A118 *Δhns* strains.

**Supplementary Table S4**

|  | MIC (ug/mL) | | | | | | | |
| --- | --- | --- | --- | --- | --- | --- | --- | --- |
|  | MEM | CAZ | IMP | CIP | NOR | COL | AN | GN |
| AB5075 | 24 | >256 | 12 | >256 | >256 | 0.5 | 64 | 192 |
| AB5075∆*hns* | 12 | >256 | 4 | >256 | >256 | 0.5 | 8 | 8 |
| A118 | 0.125 | 1.5 | 0.25 | 0.125 | 2 | 0.5 | 1.5 | 0.5 |
| A118 ∆*hns* | 0.19 | 2 | 0.19 | 0.094 | 2 | 0.5 | 0.125 | 0.094 |

MEM: meropenem; CAZ: ceftazidime; IMP: imipenem; CIP: ciprofloxacin; NOR: norfloxacin; COL: colistin; AN: amikacin; GN: gentamicin.

**Supplementary Table S4:** E-test minimal inhibitory concentration (MIC) (μg/mL) measurements were performed for meropenem (MEM), ampicillin (AMP), ceftazidime (CAZ), imipenem (IMP), ciprofloxacin (CIP), norfloxacin (NOR), polymyxin B/colistin (PB/CST), amikacin (AN) and gentamicin (GN).

**Supplementary Figure S1**

**
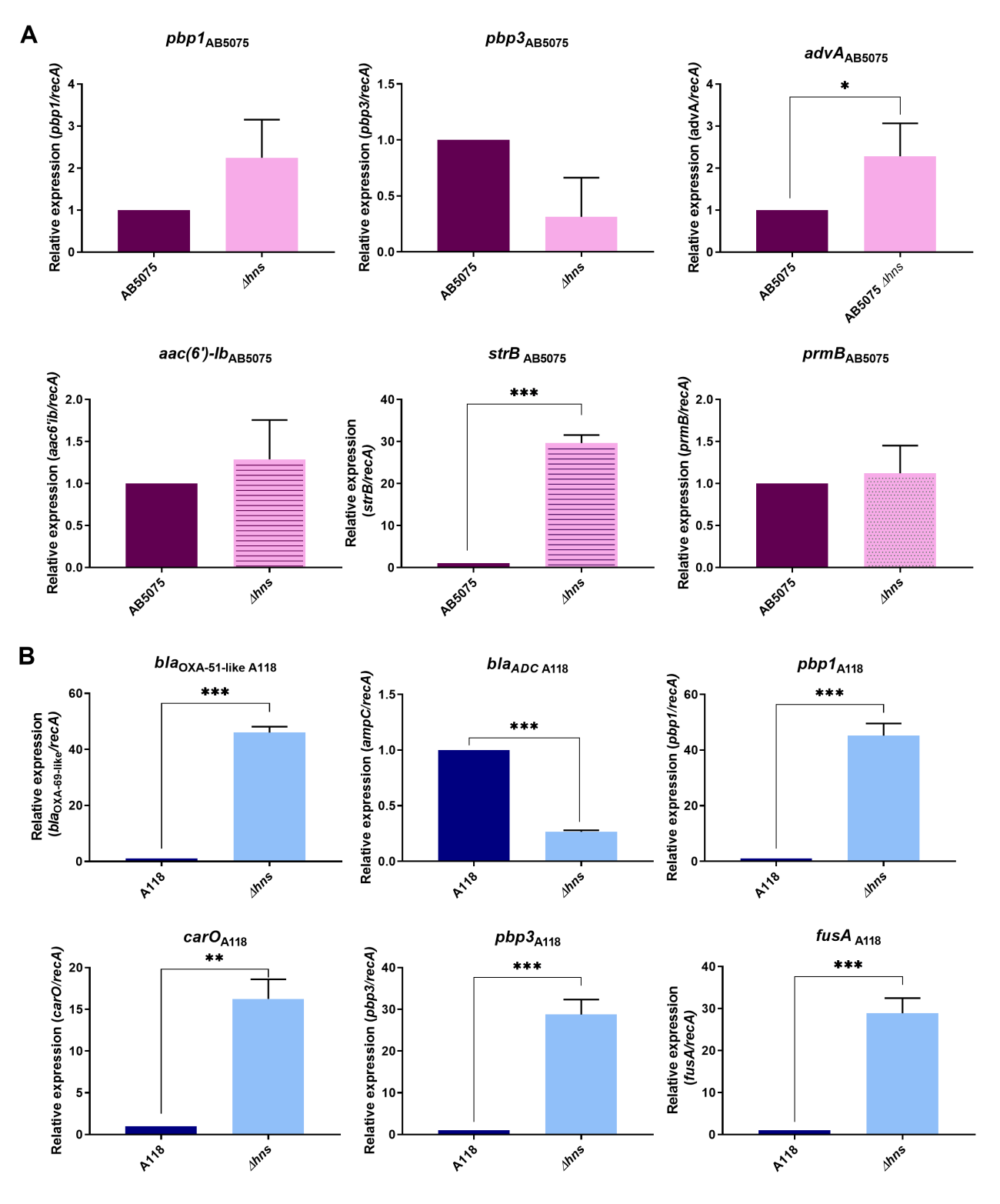
**

**Supplementary Figure S1:** Determination of relative expression levels of antibiotic resistance-associated genes in *A. baumannii* (A) AB5075 and (B) A118 *Δhns* versus parental strain. qRT-PCR was used to determine the expression levels and the fold changes used double ΔCt analysis. At least two independent samples were used, and three technical replicates were performed from each sample. Statistical significance (*P-value* < 0.05) was determined by *t* test; one asterisks: *P-value* < 0.05; two asterisks: *P-value* < 0.01 and three asterisks: *P-value* < 0.001.

**Supplementary Figure S2**


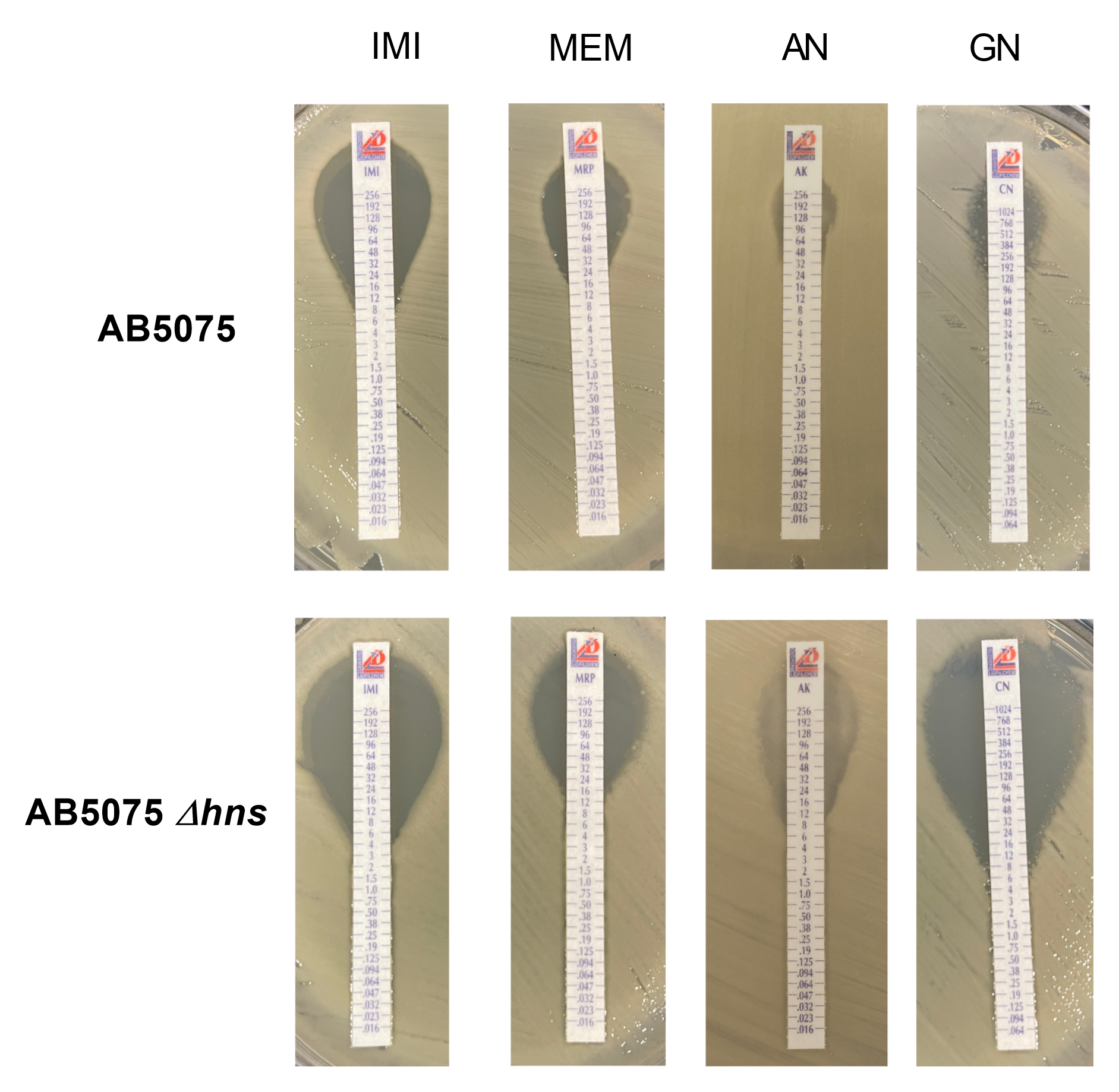


**Supplementary Figure S2:** MICs (μg/ml) of *A. baumannii* AB5075 and AB5075 *Δhns* strains

**Supplementary Figure S3**

**
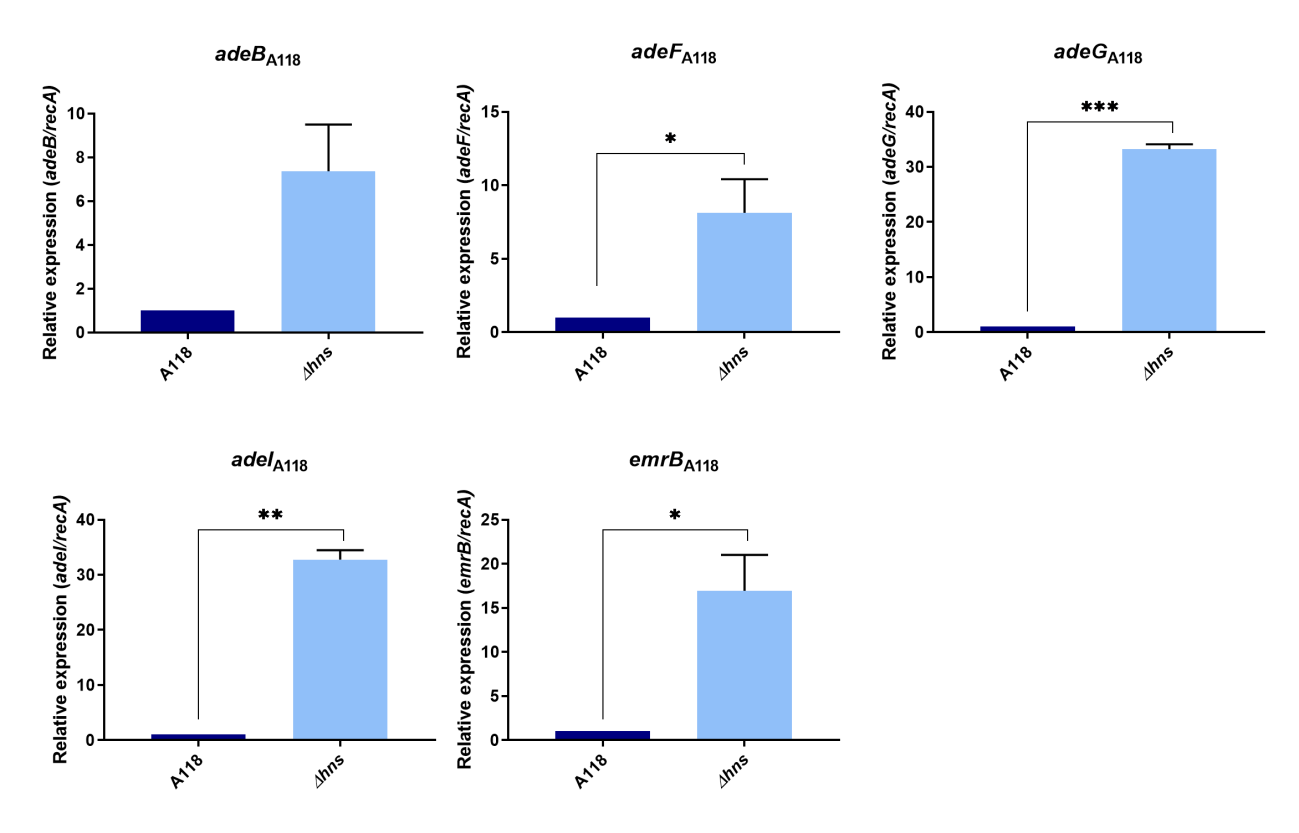
**

**Supplementary Figure S3:** Determination of relative expression levels of efflux pumps-associated genes in *A. baumannii* A118 *Δhns* vs. parental strain. qRT-PCR was used to determine the expression levels and the fold changes used double ΔCt analysis. At least two independent samples were used, and three technical replicates were performed from each sample. Statistical significance (*P-value* < 0.05) was determined by *t* test; one asterisks: *P-value* < 0.05; two asterisks: *P-value* < 0.01 and three asterisks: *P-value* < 0.001.

**Supplementary Figure S4**

**
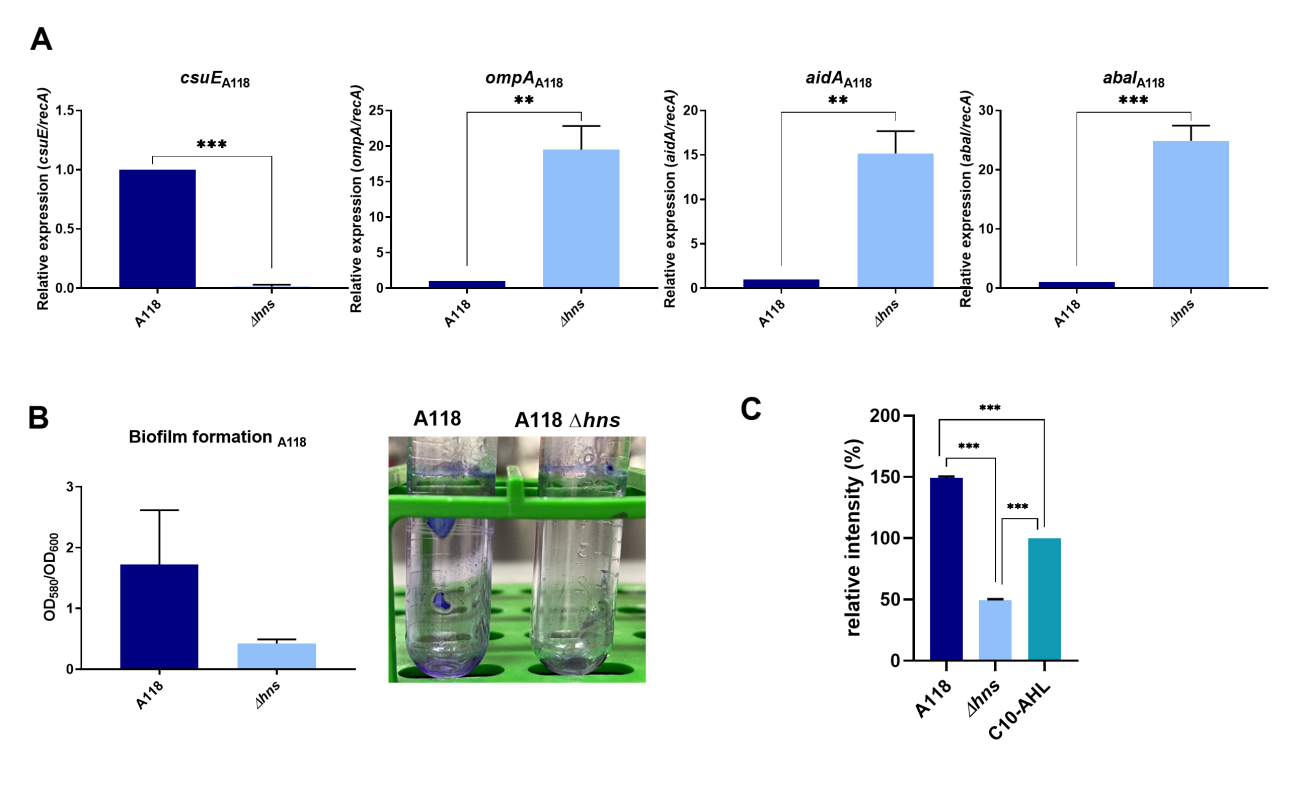
**

**Supplementary Figure S4.** Genetic and phenotypic analysis of biofilm and quorum sensing coding genes in *A. baumannii* A118 *Δhns* vs. parental strain. (A) qRT-PCR of genes associated with biofilm and quorum sensing. (B) Biofilm assays represented by OD580/OD600. Statistical analysis (*t* test) was performed and a *P-value* < 0.05 was considered significant. (C) Agar plate assay for the detection of AHL using *A. tumefaciens.* The presence of AHL was determined by the development of blue coloring. Quantification of 5,5'-dibromo-4,4'-dichloro-indigo were estimated as the percentage relative to C10-AHL standard, measured with ImageJ (NIH). The mean ± SD is informed. Statistical significance (*P-value* < 0.05) was determined by ANOVA followed by Tukey’s multiple-comparison test. Experiments were performed in triplicate, with at least three technical replicates per biological replicate.

**Supplementary Figure S5**


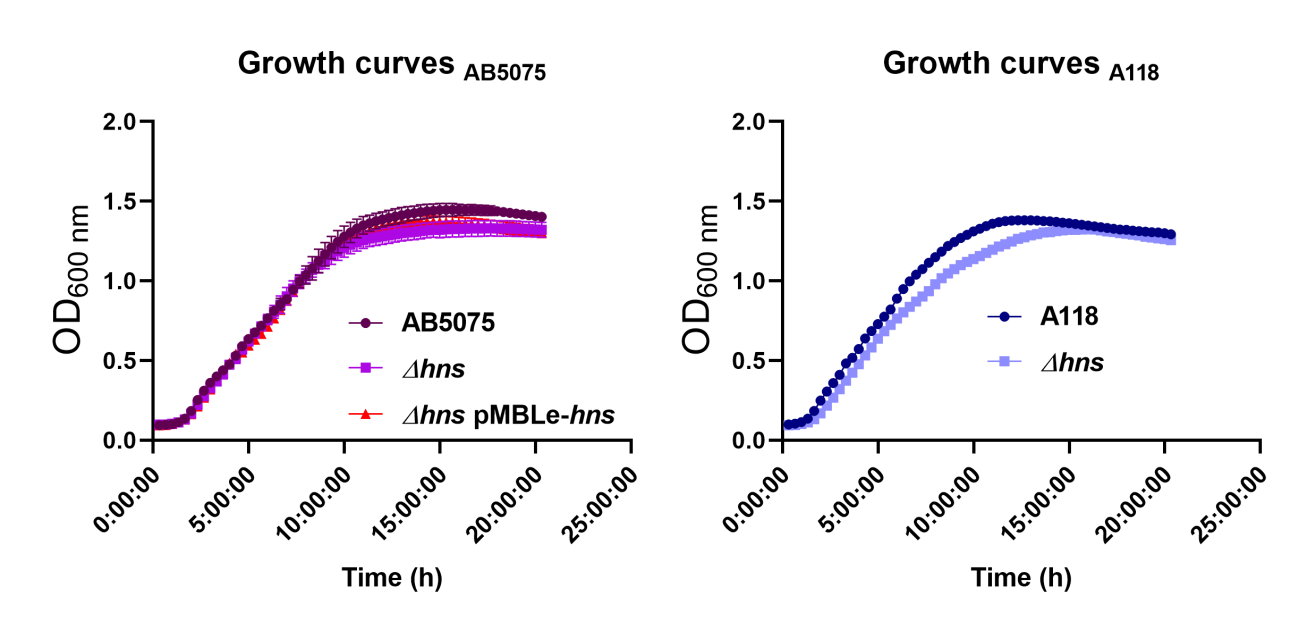


**Supplementary Figure S5.** Growth curves of *A. baumannii* AB5075 and A118 wild-type and derivative strains used in this work grown in LB broth. Optical density of 600 nm was measured using a micro-plate reader (SpectraMax M3 microplate/ cuvette reader with SoftMax Pro v6 software).
